# Crystal structure of 5-(dibenzo­furan-4-yl)-2′-deoxy­uridine

**DOI:** 10.1107/S2056989017013111

**Published:** 2017-09-19

**Authors:** Vijay Gayakhe, Anant Ramakant Kapdi, Yulia Borozdina, Carola Schulzke

**Affiliations:** a Department of Chemistry, Institute of Chemical Technology, Nathalal Parekh Road, Matunga, Mumbai 400 019, India; b Institut für Biochemie, Ernst-Moritz-Arndt Universität Greifswald, Felix-Hausdorff-Strasse 4, D-17487 Greifswald, Germany

**Keywords:** nucleoside, palladium, catalysis, uridine, Suzuki-Miyaura cross-coupling, crystal structure

## Abstract

The mol­ecule of the title compound, C_21_H_18_N_2_O_6_, has a bent rather than a linear conformation supported by three intra­molecular C—H⋯O hydrogen bonds. The packing in the crystal lattice is largely determined by inter­actions between hydrogen atoms with oxygen atom lone pairs with one mol­ecule inter­acting with neigbouring mol­ecules *via* O—H⋯O, N—H⋯O and C—H⋯O hydrogen bonds. The title compound crystallizes in the chiral ortho­rhom­bic space group *P*2_1_2_1_2_1_. Its absolute structure could not be determined crystallographically and was assumed with reference to that of the reactant 5-iodo-2′-de­oxy­uridine.

## Abstract 2

De­oxy­uridine substituted by dibenzo­furanyl at the carbon atom in base position C^5^ was synthesized and structurally characterized. The coupling was achieved by a Suzuki–Miyaura reaction utilizing the PTABS ligand and palladium(II) acetate.

## Chemical context   

As a result of their numerous applications, synthetically modified nucleoside analogues have attracted much attention in recent years. Many of these modified nucleosides show potential activity as drug candidates, biological probes *etc* (Huryn & Okabe, 1992 ▸). Modern trends in this field of research consider palladium complexes to be active catalysts for the efficient modification of nucleosides because of their greater ability to perform such catalytic processes in aqueous media (Agrofoglio *et al.*, 2003 ▸; Kapdi *et al.*, 2014 ▸). Base modification in purine and pyrimidine nucleosides, resulting in a new class of compounds with better fluorescence properties, enhancing their chances of being employed as biological probes for studying biological environments such as DNA damage, protein–DNA inter­actions and DNA probes is of great inter­est to chemical biologists as well as bio-organic chemists (Tanpure *et al.*, 2013 ▸). Structural elucidation of such compounds is an important task in order to understand the mechanistic pathways. Herein we present the synthesis and the crystal structure of the title compound, 5-(dibenzo­furan-4-yl)-2′-de­oxy­uridine.
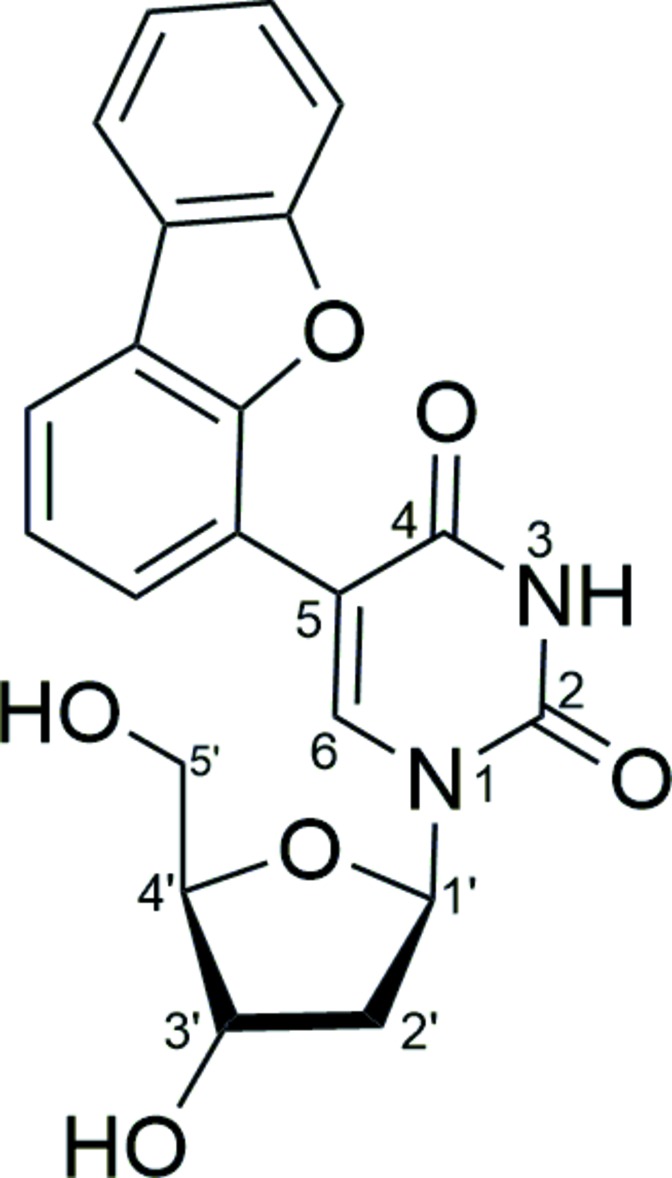


## Structural commentary   

**Figure 1:**
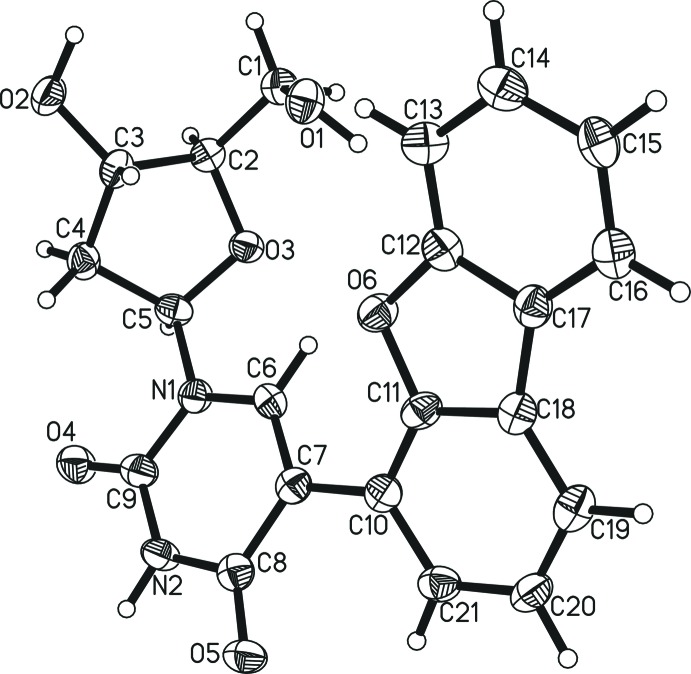
The mol­ecular structure of the title compound, showing the atom labelling and 50% probability displacement ellipsoids. Atom C7 is in the C^5^ position of the pyrimidine base according to nucleoside/nucleotide nomenclature, atom C6 in C^6^.

The title compound crystallizes in the ortho­rhom­bic space group *P*2_1_2_1_2_1_ with four mol­ecules in the unit cell. The two aromatic π systems (pyrimidine and dibenzo­furan­yl), which are connected by a C—C bond [C7—C10 = 1.489 (6) Å] subtend a dihedral angle of 30.7 (2)° (Fig. 1 ▸). All bond lengths or angles are comparable to those in related compounds. Fifty two entries can be found in the Cambridge Crystallographic Database (*ConQuest* Version 1.19; Groom *et al.*, 2016 ▸) for de­oxy­uridine with a substituent only in the C^5^ position of the base (*i.e.* C7 here) and neither substituents nor protecting groups anywhere else, nine of which are for compounds that had already been characterized (*i.e*. repeats, polymorphs, present/absent solvent). The bond lengths of the pyrimidine moiety observed for the title compound are very close to the average values found for related structures (see Table S1 in the Supporting information). As is typical for this class of compounds, the bond usually assigned to be a double bond within the six-membered ring (here C6=C7) is the shortest for the pyrimidine ring at 1.353 (6) Å and the bond between the second carbonyl carbon atom and the substituted carbon (here C7—C8) is the longest at 1.447 (6) Å. All four other ring atom-to-ring atom distances (N—C and C—C bonds) are shorter than 1.393 Å, indicating significant π-electron delocal­ization throughout the pyrimidine base. All this, however, is in accordance with the majority of previously reported structures.

The relative orientation between sugar and base moieties in the title compound is also comparable with compounds in the database. The hydrogen-bonding inter­action (or distance) between the C^6^-H function (here C6) and the ring oxygen atom of the sugar (here O3) and/or the –CH_3_–OH group (here O1) is useful for evaluation in this context. The C—H⋯O hydrogen-to-oxygen distances for the inter­action with the alcohol range from 2.29 to 5.98 Å (when the –CH_3_–OH moiety is pointing directly towards the C–H or completely turned away, respectively; Moore *et al.*, 1989 ▸; Basnak *et al.*, 1996 ▸). The C—H⋯O hydrogen-to-oxygen distances for the inter­action with the furane ring oxygen atom (here O3) range from 2.26 to 3.43 Å (Greco & Tor, 2007 ▸; Basnak *et al.*, 1996 ▸) with the vast majority of orientations allowing at least weak hydrogen bonding between this oxygen and the C^6^–H hydrogen atom. No systematic dependency between these two groups of distances was found, *i.e.* a very short or long hydrogen bond with the ring oxygen atom does neither lead to particularly short nor long distances of the hydrogen atom to the methanoyl oxygen atom.

Only five of the related archived structures bear directly attached aromatic π-systems. In all five cases, the orientation of the sugar and the pyrimidine moieties are relatively similar in which the C^6^–H moiety points to some extent towards the methanoyl oxygen atom of the sugar, forming a weak intra­molecular hydrogen bond and resulting in comparable mol­ecular bends. The dihedral angles between the two aromatic systems do vary and range from 11.9° for a ferrocene substituent (Song *et al.*, 2006 ▸) to 37.2° for a *para*-biphenyl substituent (Gayakhe *et al.*, 2016 ▸), indicating that the extent of delocalization of the π-systems depends on the actual type of aromatic substituent but is not particularly strong in any case.

## Supra­molecular Features   

**Figure 2:**
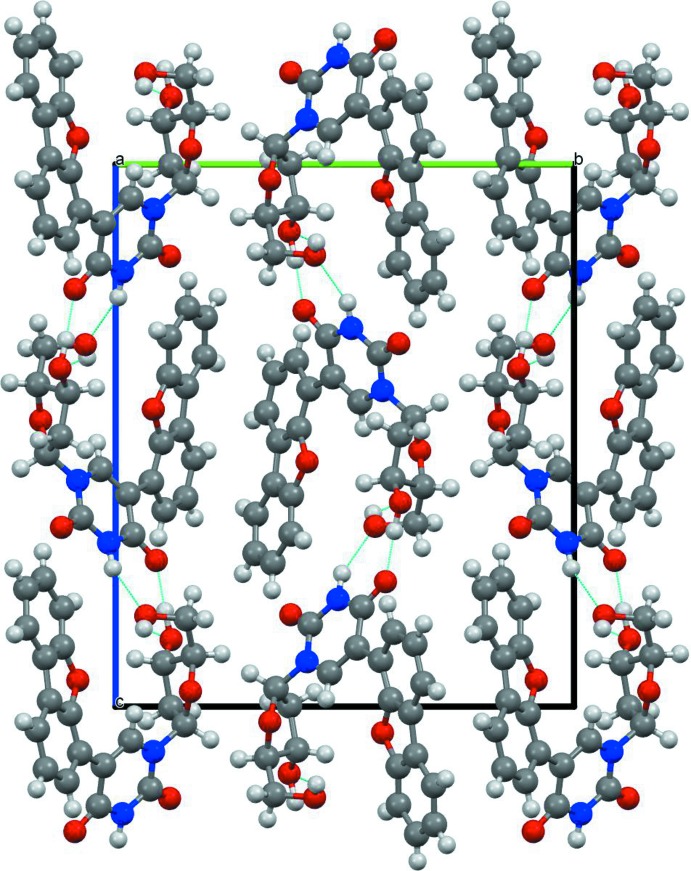
The crystal packing (*Mercury*; Macrae *et al.*, 2006 ▸) viewed along the *a* axis showing the classical hydrogen bonds which lead to a two-dimensional network parallel to (010).

In the crystal, mol­ecules are linked by N—H⋯O, O—H⋯O and C—H⋯O hydrogen bonds (Fig. 2 ▸ and Table 1 ▸). The mol­ecules form rows propagating along the *a*-axis direction, which are connected to adjacent rows in the *c*-axis direction by classical hydrogen bonds and in the *b*-axis direction only by weaker C—H⋯O contacts between two sugar moieties (C4—H4*A*⋯O3^i^, two-directional). In the *c*- and (by bifurcation) *a*-axis directions, both classical and non-classical hydrogen bonds are present (O2–H2*O*⋯O5^ii^; O1—H1*O*⋯O2^iv^; N2—H2*N*⋯O1^iii^; C13—H13⋯O4; C14—H14⋯O4^ii^). These interactions lead to the formation of slabs lying parallel to the *ac* plane.

## Synthesis and crystallization   

The title compound was synthesized according to our recently reported method (Bhilare *et al.*, 2016 ▸). This involves the cross-coupling reaction of 5-iodo-2′-deoxyuridine and 4-(dibenzofuranyl)boronic acid in the presence of Pd(OAc)_2_ and PTBS (phospha-triaza-adamantyl propane sulfonate) in water.

**Synthesis of 5-(dibenzo­furan-4-yl)-2′-de­oxy­uridine:** To a solution of palladium acetate (1.12 mg, 1.0 mol %) and PTABS ligand (2.93 mg, 2.0 mol %) in degassed water (1.0 ml) at ambient temperature under N_2_ were added 5-iodo-2′-de­oxy­uridine (0.5 mmol) and the solution stirred for 5 min at 353 K. After that, the reaction mixture was allowed to cool to room temperature and then 4-(dibenzo­furan­yl)boronic acid (0.75 mmol) was added along with tri­ethyl­amine (0.14 ml, 1.0 mmol) and degassed water (2.0 ml). The resulting solution was then stirred at 353 K for 3 h. The reaction progress was monitored by TLC. After the completion of reaction, the solvent was removed *in vacuo* and the resultant residue obtained was purified using column chromatography in CH_2_Cl_2_:MeOH solvent system (96:4) to afford the desired product as a white solid (162 mg, 82% yield).

UV–visible absorption and fluorescence emission in methanol (10 µ*M*) λ_abs_ = 286 nm λ_fl_ = 392,427. ^1^H NMR (400 MHz, DMSO-*d*_6_) δ 11.62 (*s*, 1H), 8.41 (*s*, 1H), 8.12 (*d*, *J* = 7.4 Hz, 1H), 8.06 (*d*, *J* = 7.7 Hz, 1H), 7.67 (*t*, *J* = 7.8 Hz, 2H), 7.49 (*t*, *J* = 7.7 Hz, 1H), 7.38 (*t*, *J* = 7.6 Hz, 2H), 6.28 (*t*, *J* = 6.7 Hz, 1H), 5.29 (*d*, *J* = 3.8 Hz, 1H), 4.87 (*t*, *J* = 4.9 Hz, 1H), 4.27 (*s*, 1H), 3.81 (*d*, *J* = 2.9 Hz, 1H), 3.54 (*s*, 2H), 2.29–2.14 (*m*, 2H). ^13^C NMR (101 MHz, DMSO-*d*_6_) δ 161.7, 155.3, 152.9, 150.0, 140.1, 128.3, 127.6, 123.8, 123.6, 123.2, 122.8, 121.1, 120.3, 117.8, 111.7, 108.8, 87.6, 84.5, 70.5, 61.4, 39.9. ESI–MS (*m*/*z*) = 395 (*M* ^+^ + H^+^). Analysis calculated for C_21_H_18_N_2_O_6_: C, 63.96; H, 4.60; N, 7.10. Found: C, 63.85; H, 4.64; N, 6.98.

## Refinement   

Crystal data, data collection and structure refinement details are summarized in Table 2 ▸. The two protons on oxygen (O1, O2) and the one on nitro­gen (N2) were located and refined with a constraint for the atom—H distance (*SHELXL* instruction: SADI 0.05 O1 H1*O* O2 H2*O* N2 H2*N*), as otherwise the N—H distance became rather short and the O—H distances rather long. The respective orientations, *i.e.* the directions the hydrogen atoms are pointing to (particularly important for the alcohol functions), were refined without any restraints or constraints. The C-bound H atoms were included in calculated positions and treated as riding: C—H = 0.95–1.00 Å with *U*_iso_(H) = 1.2*U*_eq_(C).

## Supplementary Material

Crystal structure: contains datablock(s) I, global. DOI: 10.1107/S2056989017013111/ds2247sup1.cif

Structure factors: contains datablock(s) I. DOI: 10.1107/S2056989017013111/ds2247Isup2.hkl

Bond lengths in uracil bases with substitution at the C5 position. DOI: 10.1107/S2056989017013111/ds2247sup3.pdf

CCDC reference: 1574284

Additional supporting information:  crystallographic information; 3D view; checkCIF report

## Figures and Tables

**Table 1:** Hydrogen-bond geometry (Å, °)

*D*—H⋯*A*	*D*—H	H⋯*A*	*D*⋯*A*	*D*—H⋯*A*
C4—H4*A*⋯O3^i^	0.99	2.61	3.439 (6)	142
C6—H6⋯O6	0.95	2.34	2.915 (5)	119
C13—H13⋯O1	0.95	2.58	3.271 (6)	130
C21—H21⋯O5	0.95	2.33	2.876 (6)	116
C14—H14⋯O4^ii^	0.95	2.45	3.115 (6)	127
O2—H2*O*⋯O5^ii^	1.00 (5)	1.72 (5)	2.716 (5)	174 (5)
N2—H2*N*⋯O1^iii^	0.91 (5)	2.30 (5)	3.144 (5)	154 (5)
O1—H1*O*⋯O2^iv^	0.92 (6)	2.10 (6)	2.922 (5)	148 (6)

Symmetry codes: (i) 
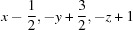
; (ii) 
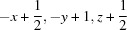
; (iii) 
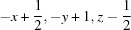
; (iv) 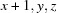.

**Table 2:** Experimental details

Crystal data	
Chemical formula	C_21_H_18_N_2_O_6_
*M*_r_	394.37
Crystal system, space group	Orthorhombic, *P*2_1_2_1_2_1_
Temperature (K)	170
*a*, *b*, *c* (Å)	6.2899 (13), 15.167 (3), 17.938 (4)
*V* (Å^3^)	1711.2 (6)
*Z*	4
Radiation type	Mo *K*α
μ (mm^−1^)	0.11
Crystal size (mm)	0.46 × 0.09 × 0.09

Data collection	
Diffractometer	Stoe IPDS2T
Absorption correction	Numerical face indexed (*X-RED32* and *X-SHAPE*; Stoe & Cie, 2010 ▸)
*T*_min_, *T*_max_	0.388, 0.875
No. of measured, independent and observed [*I* > 2σ(*I*)] reflections	14640, 3696, 2704
*R*_int_	0.110
(sin θ/λ)_max_ (Å^−1^)	0.642

Refinement	
*R*[*F* ^2^ > 2σ(*F* ^2^)], *wR*(*F* ^2^), *S*	0.057, 0.143, 0.96
No. of reflections	3696
No. of parameters	274
No. of restraints	3
H-atom treatment	H atoms treated by a mixture of independent and constrained refinement
Δρ_max_, Δρ_min_ (e Å^−3^)	0.34, −0.37

Computer programs: *X-AREA* (Stoe & Cie, 2010 ▸), *SHELXT2014* (Sheldrick, 2015*a* ▸), *SHELXL2013* (Sheldrick, 2015*b* ▸), *XP* in *SHELXTL* and *CIFTAB* (Sheldrick, 2008 ▸), *Mercury* (Macrae *et al.*, 2006 ▸) and *PLATON* (Spek, 2009 ▸).

## supplementary crystallographic information

### Crystal data

**Table:**

C_21_H_18_N_2_O_6_	*D*_x_ = 1.531 Mg m^−^^3^
*M**_r_* = 394.37	Mo *K*α radiation, λ = 0.71073 Å
Orthorhombic, *P*2_1_2_1_2_1_	Cell parameters from 14682 reflections
*a* = 6.2899 (13) Å	θ = 6.5–54.3°
*b* = 15.167 (3) Å	µ = 0.11 mm^−^^1^
*c* = 17.938 (4) Å	*T* = 170 K
*V* = 1711.2 (6) Å^3^	Needle, colourless
*Z* = 4	0.46 × 0.09 × 0.09 mm
*F*(000) = 824	

### Data collection

**Table:**

Stoe IPDS2T diffractometer	3696 independent reflections
Radiation source: fine-focus sealed tube	2704 reflections with *I* > 2σ(*I*)
Detector resolution: 6.67 pixels mm^-1^	*R*_int_ = 0.110
ω scans	θ_max_ = 27.1°, θ_min_ = 3.4°
Absorption correction: numerical face indexed (X-Red32 and X-Shape; Stoe & Cie, 2010)	*h* = −7→8
*T*_min_ = 0.388, *T*_max_ = 0.875	*k* = −19→19
14640 measured reflections	*l* = −22→22

### Refinement

**Table:**

Refinement on *F*^2^	3 restraints
Least-squares matrix: full	Hydrogen site location: mixed
*R*[*F*^2^ > 2σ(*F*^2^)] = 0.057	H atoms treated by a mixture of independent and constrained refinement
*wR*(*F*^2^) = 0.143	*w* = 1/[σ^2^(*F*_o_^2^) + (0.0841*P*)^2^] where *P* = (*F*_o_^2^ + 2*F*_c_^2^)/3
*S* = 0.96	(Δ/σ)_max_ < 0.001
3696 reflections	Δρ_max_ = 0.34 e Å^−^^3^
274 parameters	Δρ_min_ = −0.37 e Å^−^^3^

### Special details

**Table:**

Experimental. The reaction was carried out in a Schlenk tube using Schlenk techniques under a nitrogen atmosphere. All other reagents and solvents were purchased commercially and used without any further purification. A UV–visible spectrum of the title compound (10 µ*M*) was measured in methanol using a UV–visible spectrophotometer with a cell of 1 cm path length. A fluorescence spectrum of the same solution was obtained using a fluorescence spectrophotometer at 298 K using a 1 cm path-length cell. The reaction was monitored by thin layer chromatography using TLC silica gel 60 F254 precoated plates (Merck). Visualization was accomplished by irradiation with UV light. C, H, and N analyses was carried out locally. NMR data (^1^H, ^13^C) of the synthesized compound were recorded locally on 500 MHz spectrometers. Mass spectroscopic analysis was carried out with a mass spectrometer from Varian Inc, US: 10 Prostar Binary LC with 500 MS IT PDA detectors.
Geometry. All esds (except the esd in the dihedral angle between two l.s. planes) are estimated using the full covariance matrix. The cell esds are taken into account individually in the estimation of esds in distances, angles and torsion angles; correlations between esds in cell parameters are only used when they are defined by crystal symmetry. An approximate (isotropic) treatment of cell esds is used for estimating esds involving l.s. planes.

### Fractional atomic coordinates and isotropic or equivalent isotropic displacement parameters (Å^2^)

**Table:**

	*x*	*y*	*z*	*U*_iso_*/*U*_eq_	
O1	0.4367 (6)	0.5659 (2)	0.66750 (19)	0.0316 (8)	
O2	−0.1365 (5)	0.6212 (3)	0.62349 (19)	0.0355 (8)	
O3	0.3555 (5)	0.6634 (2)	0.52801 (16)	0.0251 (7)	
O4	0.0678 (6)	0.6179 (2)	0.33056 (18)	0.0342 (8)	
O5	0.5541 (6)	0.4112 (2)	0.26958 (17)	0.0319 (8)	
O6	0.6845 (5)	0.4177 (2)	0.54844 (17)	0.0271 (7)	
N1	0.3167 (6)	0.5825 (2)	0.4169 (2)	0.0244 (8)	
N2	0.3171 (7)	0.5149 (3)	0.3022 (2)	0.0278 (8)	
C1	0.3630 (8)	0.6543 (3)	0.6624 (2)	0.0268 (10)	
H1A	0.4869	0.6946	0.6612	0.032*	
H1B	0.2787	0.6685	0.7074	0.032*	
C2	0.2282 (7)	0.6702 (3)	0.5941 (2)	0.0238 (9)	
H2	0.1672	0.7310	0.5970	0.029*	
C3	0.0472 (7)	0.6046 (3)	0.5800 (2)	0.0256 (9)	
H3	0.0978	0.5428	0.5880	0.031*	
C4	0.0039 (7)	0.6206 (3)	0.4983 (3)	0.0264 (9)	
H4A	−0.1046	0.6672	0.4915	0.032*	
H4B	−0.0458	0.5660	0.4735	0.032*	
C5	0.2197 (7)	0.6500 (3)	0.4670 (2)	0.0253 (9)	
H5	0.2020	0.7067	0.4391	0.030*	
C6	0.4901 (7)	0.5330 (3)	0.4377 (2)	0.0233 (9)	
H6	0.5481	0.5409	0.4862	0.028*	
C7	0.5806 (7)	0.4735 (3)	0.3914 (2)	0.0239 (9)	
C8	0.4944 (7)	0.4627 (3)	0.3171 (3)	0.0262 (9)	
C9	0.2231 (8)	0.5755 (3)	0.3485 (2)	0.0282 (10)	
C10	0.7714 (7)	0.4220 (3)	0.4143 (2)	0.0250 (9)	
C11	0.8132 (7)	0.4001 (3)	0.4874 (2)	0.0256 (10)	
C12	0.7864 (8)	0.3833 (3)	0.6103 (2)	0.0271 (10)	
C13	0.7096 (8)	0.3830 (3)	0.6814 (3)	0.0296 (10)	
H13	0.5767	0.4090	0.6935	0.036*	
C14	0.8351 (8)	0.3429 (3)	0.7351 (3)	0.0321 (10)	
H14	0.7868	0.3412	0.7853	0.039*	
C15	1.0316 (8)	0.3047 (3)	0.7172 (3)	0.0310 (10)	
H15	1.1139	0.2775	0.7552	0.037*	
C16	1.1061 (8)	0.3060 (3)	0.6454 (3)	0.0306 (10)	
H16	1.2393	0.2802	0.6334	0.037*	
C17	0.9829 (7)	0.3460 (3)	0.5904 (3)	0.0262 (9)	
C18	0.9993 (7)	0.3562 (3)	0.5105 (3)	0.0250 (9)	
C19	1.1478 (7)	0.3293 (3)	0.4577 (3)	0.0280 (10)	
H19	1.2741	0.2993	0.4720	0.034*	
C20	1.1057 (7)	0.3474 (3)	0.3848 (3)	0.0294 (10)	
H20	1.2028	0.3278	0.3477	0.035*	
C21	0.9242 (8)	0.3939 (3)	0.3626 (3)	0.0281 (10)	
H21	0.9041	0.4067	0.3112	0.034*	
H2O	−0.097 (9)	0.608 (4)	0.676 (3)	0.037 (15)*	
H2N	0.266 (9)	0.506 (4)	0.255 (3)	0.034 (14)*	
H1O	0.567 (10)	0.562 (5)	0.644 (4)	0.07 (2)*	

### Atomic displacement parameters (Å^2^)

**Table:**

	*U*^11^	*U*^22^	*U*^33^	*U*^12^	*U*^13^	*U*^23^
O1	0.0278 (19)	0.0285 (17)	0.0385 (19)	0.0033 (13)	−0.0009 (14)	0.0040 (14)
O2	0.0208 (17)	0.054 (2)	0.0320 (19)	0.0045 (15)	0.0025 (13)	0.0041 (16)
O3	0.0267 (17)	0.0264 (15)	0.0223 (16)	−0.0019 (12)	0.0004 (12)	−0.0025 (12)
O4	0.0333 (19)	0.0388 (18)	0.0305 (17)	0.0095 (15)	−0.0064 (14)	−0.0014 (15)
O5	0.0370 (19)	0.0331 (17)	0.0255 (16)	0.0016 (14)	0.0004 (14)	−0.0086 (14)
O6	0.0269 (17)	0.0286 (16)	0.0259 (15)	0.0040 (13)	0.0028 (13)	0.0018 (12)
N1	0.024 (2)	0.0249 (18)	0.0243 (18)	0.0037 (15)	−0.0004 (15)	0.0000 (15)
N2	0.033 (2)	0.0299 (19)	0.0205 (19)	0.0018 (16)	−0.0034 (16)	−0.0039 (15)
C1	0.029 (2)	0.024 (2)	0.028 (2)	−0.0031 (18)	0.0007 (18)	−0.0005 (18)
C2	0.025 (2)	0.021 (2)	0.025 (2)	0.0013 (16)	0.0016 (18)	0.0007 (17)
C3	0.022 (2)	0.023 (2)	0.031 (2)	0.0002 (17)	0.0024 (17)	0.0028 (18)
C4	0.022 (2)	0.026 (2)	0.031 (2)	0.0039 (17)	−0.0022 (19)	−0.0032 (18)
C5	0.032 (2)	0.021 (2)	0.023 (2)	0.0024 (17)	−0.0011 (18)	−0.0010 (17)
C6	0.024 (2)	0.023 (2)	0.023 (2)	−0.0023 (16)	−0.0033 (16)	−0.0002 (16)
C7	0.024 (2)	0.021 (2)	0.026 (2)	−0.0018 (16)	0.0020 (17)	0.0012 (17)
C8	0.027 (2)	0.024 (2)	0.027 (2)	−0.0021 (17)	0.0014 (18)	0.0015 (17)
C9	0.032 (3)	0.030 (2)	0.023 (2)	−0.002 (2)	−0.0005 (18)	−0.0014 (18)
C10	0.026 (2)	0.020 (2)	0.029 (2)	−0.0015 (17)	0.0010 (18)	−0.0024 (18)
C11	0.027 (3)	0.020 (2)	0.029 (2)	0.0003 (17)	0.0060 (18)	−0.0031 (16)
C12	0.029 (2)	0.023 (2)	0.030 (2)	0.0016 (18)	−0.0041 (19)	0.0003 (18)
C13	0.036 (3)	0.023 (2)	0.030 (2)	0.0030 (19)	0.001 (2)	0.0011 (18)
C14	0.040 (3)	0.027 (2)	0.029 (2)	−0.004 (2)	−0.003 (2)	−0.0001 (19)
C15	0.030 (3)	0.028 (2)	0.036 (3)	0.0010 (19)	−0.009 (2)	0.0030 (19)
C16	0.030 (3)	0.022 (2)	0.040 (3)	0.0015 (17)	−0.006 (2)	−0.004 (2)
C17	0.024 (2)	0.023 (2)	0.032 (2)	−0.0019 (17)	−0.0003 (19)	−0.0024 (18)
C18	0.025 (2)	0.019 (2)	0.031 (2)	−0.0012 (17)	0.0012 (18)	0.0005 (17)
C19	0.026 (2)	0.017 (2)	0.041 (3)	0.0010 (16)	0.004 (2)	−0.0001 (18)
C20	0.027 (2)	0.029 (2)	0.033 (3)	0.0008 (18)	0.0083 (19)	−0.0053 (19)
C21	0.033 (3)	0.025 (2)	0.026 (2)	−0.0032 (18)	0.0028 (19)	−0.0045 (18)

### Geometric parameters (Å, º)

**Table:**

O1—C1	1.423 (6)	C5—H5	1.0000
O1—H1O	0.92 (6)	C6—C7	1.353 (6)
O2—C3	1.417 (6)	C6—H6	0.9500
O2—H2O	1.00 (5)	C7—C8	1.447 (6)
O3—C5	1.403 (5)	C7—C10	1.489 (6)
O3—C2	1.435 (5)	C10—C11	1.379 (6)
O4—C9	1.213 (6)	C10—C21	1.402 (6)
O5—C8	1.216 (5)	C11—C18	1.408 (6)
O6—C12	1.384 (5)	C12—C13	1.364 (7)
O6—C11	1.387 (5)	C12—C17	1.405 (6)
N1—C9	1.366 (6)	C13—C14	1.386 (7)
N1—C6	1.375 (6)	C13—H13	0.9500
N1—C5	1.492 (5)	C14—C15	1.403 (7)
N2—C9	1.373 (6)	C14—H14	0.9500
N2—C8	1.393 (6)	C15—C16	1.370 (7)
N2—H2N	0.91 (5)	C15—H15	0.9500
C1—C2	1.508 (6)	C16—C17	1.394 (6)
C1—H1A	0.9900	C16—H16	0.9500
C1—H1B	0.9900	C17—C18	1.445 (6)
C2—C3	1.533 (6)	C18—C19	1.391 (6)
C2—H2	1.0000	C19—C20	1.364 (7)
C3—C4	1.511 (6)	C19—H19	0.9500
C3—H3	1.0000	C20—C21	1.399 (7)
C4—C5	1.535 (7)	C20—H20	0.9500
C4—H4A	0.9900	C21—H21	0.9500
C4—H4B	0.9900		
			
C1—O1—H1O	109 (5)	C6—C7—C10	121.3 (4)
C3—O2—H2O	106 (3)	C8—C7—C10	119.8 (4)
C5—O3—C2	108.4 (3)	O5—C8—N2	118.5 (4)
C12—O6—C11	106.8 (3)	O5—C8—C7	127.0 (4)
C9—N1—C6	122.9 (4)	N2—C8—C7	114.4 (4)
C9—N1—C5	114.7 (4)	O4—C9—N1	122.9 (4)
C6—N1—C5	122.4 (4)	O4—C9—N2	122.8 (4)
C9—N2—C8	127.5 (4)	N1—C9—N2	114.2 (4)
C9—N2—H2N	121 (4)	C11—C10—C21	115.1 (4)
C8—N2—H2N	112 (4)	C11—C10—C7	122.8 (4)
O1—C1—C2	112.7 (4)	C21—C10—C7	122.0 (4)
O1—C1—H1A	109.1	C10—C11—O6	126.3 (4)
C2—C1—H1A	109.1	C10—C11—C18	123.5 (4)
O1—C1—H1B	109.1	O6—C11—C18	110.2 (4)
C2—C1—H1B	109.1	C13—C12—O6	125.9 (4)
H1A—C1—H1B	107.8	C13—C12—C17	123.2 (4)
O3—C2—C1	110.2 (4)	O6—C12—C17	110.8 (4)
O3—C2—C3	103.3 (3)	C12—C13—C14	116.8 (5)
C1—C2—C3	116.7 (4)	C12—C13—H13	121.6
O3—C2—H2	108.8	C14—C13—H13	121.6
C1—C2—H2	108.8	C13—C14—C15	121.5 (5)
C3—C2—H2	108.8	C13—C14—H14	119.2
O2—C3—C4	111.0 (4)	C15—C14—H14	119.2
O2—C3—C2	113.5 (4)	C16—C15—C14	120.8 (5)
C4—C3—C2	101.0 (4)	C16—C15—H15	119.6
O2—C3—H3	110.4	C14—C15—H15	119.6
C4—C3—H3	110.4	C15—C16—C17	118.8 (5)
C2—C3—H3	110.4	C15—C16—H16	120.6
C3—C4—C5	104.0 (4)	C17—C16—H16	120.6
C3—C4—H4A	111.0	C16—C17—C12	118.9 (4)
C5—C4—H4A	111.0	C16—C17—C18	135.2 (4)
C3—C4—H4B	111.0	C12—C17—C18	105.8 (4)
C5—C4—H4B	111.0	C19—C18—C11	119.8 (4)
H4A—C4—H4B	109.0	C19—C18—C17	133.7 (4)
O3—C5—N1	108.6 (3)	C11—C18—C17	106.4 (4)
O3—C5—C4	107.2 (3)	C20—C19—C18	117.6 (4)
N1—C5—C4	112.5 (4)	C20—C19—H19	121.2
O3—C5—H5	109.5	C18—C19—H19	121.2
N1—C5—H5	109.5	C19—C20—C21	122.2 (4)
C4—C5—H5	109.5	C19—C20—H20	118.9
C7—C6—N1	122.1 (4)	C21—C20—H20	118.9
C7—C6—H6	118.9	C20—C21—C10	121.7 (4)
N1—C6—H6	118.9	C20—C21—H21	119.2
C6—C7—C8	118.8 (4)	C10—C21—H21	119.2

### Hydrogen-bond geometry (Å, º)

**Table:**

*D*—H···*A*	*D*—H	H···*A*	*D*···*A*	*D*—H···*A*
C4—H4*A*···O3^i^	0.99	2.61	3.439 (6)	142
C6—H6···O6	0.95	2.34	2.915 (5)	119
C13—H13···O1	0.95	2.58	3.271 (6)	130
C21—H21···O5	0.95	2.33	2.876 (6)	116
C13—H13···O4^ii^	0.95	2.65	3.194 (6)	117
C14—H14···O4^ii^	0.95	2.45	3.115 (6)	127
C1—H1*B*···O5^ii^	0.99	2.66	3.401 (6)	132
O2—H2*O*···O5^ii^	1.00 (5)	1.72 (5)	2.716 (5)	174 (5)
N2—H2*N*···O1^iii^	0.91 (5)	2.30 (5)	3.144 (5)	154 (5)
O1—H1*O*···O2^iv^	0.92 (6)	2.10 (6)	2.922 (5)	148 (6)

Symmetry codes: (i) *x*−1/2, −*y*+3/2, −*z*+1; (ii) −*x*+1/2, −*y*+1, *z*+1/2; (iii) −*x*+1/2, −*y*+1, *z*−1/2; (iv) *x*+1, *y*, *z*.
