# Supplementary material for: Crystal structure of 5-(dibenzo­furan-4-yl)-2′-deoxy­uridine
Source: Acta Crystallogr E Crystallogr Commun. 2017 Sep 19;73(Pt 10):1493–6. doi: 10.1107/S2056989017013111 (PMC5730302; doi:10.1107/S2056989017013111)
Supplement: Supplementary file 3 [file e-73-01493-sup3.pdf]

Table S1: Bond lengths of uracil bases with substitution in C^5 position (including thymin) as available in the Cambridge crystallographic database and bond lengths found in the novel compound presented here.

	N1-> CO	CO-> NH	NH-> CO	CO-> C-C	C-C -> CH	CH -> N1	C=O 1	C=O 2
EWALAR	1,389	1,377	1,377	1,447	1,357	1,363	1,216	1,233
ACDXUR	1,381	1,377	1,393	1,456	1,369	1,348	1,219	1,225
ACURID	1,406	1,367	1,400	1,460	1,359	1,362	1,215	1,223
CABSIH10	1,386	1,374	1,373	1,460	1,341	1,385	1,210	1,229
CASKAI	1,383	1,367	1,371	1,471	1,337	1,371	1,219	1,217
	1,376	1,361	1,371	1,484	1,345	1,343	1,206	1,223
CUKHEW	1,376	1,368	1,382	1,456	1,357	1,373	1,224	1,227
	1,384	1,365	1,382	1,452	1,356	1,365	1,223	1,228
DISKEW	1,387	1,367	1,389	1,457	1,351	1,363	1,220	1,223
DUHHUJ	1,391	1,384	1,372	1,451	1,347	1,367	1,205	1,228
ETYXUR	1,388	1,373	1,375	1,452	1,358	1,369	1,219	1,225
FATTOJ	1,379	1,381	1,373	1,449	1,339	1,382	1,206	1,227
FIHXID	1,373	1,375	1,368	1,467	1,347	1,373	1,220	1,223
FMDURD01	1,379	1,415	1,379	1,438	1,349	1,383	1,177	1,223
FMDURD02	1,388	1,378	1,375	1,439	1,340	1,360	1,207	1,227
FUWHAH	1,387	1,386	1,395	1,458	1,362	1,361	1,219	1,245
	1,405	1,386	1,372	1,457	1,329	1,412	1,192	1,246
FUWHEL	1,382	1,378	1,384	1,452	1,363	1,377	1,221	1,232
HEZWEP	1,374	1,365	1,395	1,458	1,363	1,372	1,225	1,219
	1,373	1,359	1,387	1,457	1,355	1,371	1,236	1,224
HEZWIT	1,388	1,369	1,385	1,455	1,353	1,365	1,221	1,228
	1,393	1,376	1,381	1,461	1,354	1,364	1,213	1,224
HEZWOZ	1,364	1,365	1,375	1,447	1,345	1,372	1,237	1,225
	1,364	1,360	1,387	1,436	1,353	1,359	1,239	1,220
HIFVEY	1,377	1,373	1,392	1,449	1,355	1,369	1,222	1,227
	1,384	1,373	1,385	1,456	1,344	1,371	1,220	1,230
HMDOUR	1,374	1,377	1,379	1,448	1,356	1,354	1,214	1,222
HOLSUY	1,379	1,376	1,385	1,459	1,351	1,367	1,216	1,236
IJIHIU	1,390	1,373	1,374	1,451	1,355	1,369	1,202	1,223
IJIHOA	1,382	1,377	1,374	1,449	1,351	1,363	1,208	1,236
IJIHUG	1,377	1,373	1,378	1,458	1,351	1,375	1,218	1,222
IPDXUR	1,384	1,360	1,397	1,458	1,343	1,401	1,227	1,226
KAMCUW	1,370	1,371	1,386	1,447	1,349	1,396	1,231	1,219
KIYFAZ	1,377	1,355	1,369	1,416	1,334	1,377	1,203	1,246
MOLQUZ	1,355	1,377	1,368	1,456	1,347	1,386	1,225	1,213
	1,392	1,346	1,376	1,451	1,332	1,379	1,236	1,236
MOLREK	1,380	1,372	1,365	1,418	1,303	1,381	1,208	1,276
	1,386	1,348	1,354	1,455	1,370	1,362	1,217	1,199
NEKBOV	1,377	1,376	1,387	1,455	1,343	1,372	1,221	1,220
PULVIB	1,374	1,378	1,383	1,447	1,360	1,374	1,216	1,233
REMSOR	1,380	1,375	1,374	1,465	1,340	1,370	1,213	1,235
SERQUC	1,380	1,366	1,386	1,442	1,348	1,364	1,233	1,234
SIFKEX	1,388	1,374	1,379	1,436	1,351	1,375	1,215	1,232
THYDIN	1,385	1,381	1,378	1,453	1,343	1,374	1,206	1,230
THYDIN01	1,390	1,367	1,368	1,446	1,341	1,370	1,212	1,226
THYDIN02	1,390	1,375	1,385	1,453	1,350	1,377	1,224	1,236
THYDIN03	1,394	1,380	1,389	1,461	1,354	1,385	1,229	1,238
THYDIN04	1,391	1,388	1,395	1,461	1,366	1,394	1,239	1,247
THYDIN05	1,387	1,374	1,377	1,452	1,343	1,378	1,213	1,228
THYMDN01	1,380	1,382	1,392	1,455	1,362	1,389	1,237	1,242
	1,379	1,382	1,393	1,455	1,358	1,392	1,240	1,241
TUFFUV	1,374	1,374	1,383	1,443	1,335	1,372	1,219	1,238
	1,378	1,371	1,393	1,450	1,352	1,367	1,215	1,216
TUFGAC	1,416	1,381	1,436	1,363	1,380	1,301	1,233	1,237
	1,276	1,405	1,393	1,443	1,428	1,328	1,224	1,183
	1,460	1,356	1,368	1,497	1,312	1,400	1,227	1,181
	1,383	1,345	1,384	1,420	1,321	1,457	1,220	1,179
TUFGEG	1,368	1,386	1,364	1,425	1,343	1,370	1,208	1,246
	1,377	1,382	1,349	1,466	1,351	1,334	1,198	1,230
VDURID	1,382	1,372	1,355	1,473	1,355	1,358	1,216	1,235
WEXGOX	1,400	1,325	1,401	1,480	1,356	1,368	1,243	1,208
	1,412	1,339	1,400	1,490	1,324	1,366	1,202	1,181
ZEMREO	1,374	1,364	1,394	1,457	1,338	1,386	1,225	1,217
Maximum	1,460	1,415	1,436	1,497	1,428	1,457	1,243	1,276
Minimum	1,276	1,325	1,349	1,363	1,303	1,301	1,177	1,179
**Average**	**1,383**	**1,372**	**1,381**	**1,452**	**1,350**	**1,372**	**1,218**	**1,226**
VGC-36 (this work)	1,367	1,373	1,392	1,448	1,353	1,375	1,212	1,216
